# Diabetes and its drivers: the largest epidemic in human history?

**DOI:** 10.1186/s40842-016-0039-3

**Published:** 2017-01-18

**Authors:** Paul Z. Zimmet

**Affiliations:** Monash University & Baker IDI Heart and Diabetes Institute, Melbourne, VIC Australia

**Keywords:** Global diabetes epidemic, Drivers for diabetes, Epigenetics and diabetes

## Abstract

The “Diabesity” epidemic (obesity and type 2 diabetes) is likely to be the biggest epidemic in human history. Diabetes has been seriously underrated as a global public health issue and the world can no longer ignore “the rise and rise” of type 2 diabetes. Currently, most of the national and global diabetes estimates come from the IDF Atlas. These estimates have significant limitations from a public health perspective. It is apparent that the IDF have consistently underestimated the global burden. More reliable estimates of the future burden of diabetes are urgently needed.

To prevent type 2 diabetes, a better understanding of the drivers of the epidemic is needed. While for years, there has been comprehensive attention to the “traditional” risk factors for type 2 diabetes i.e., genes, lifestyle and behavioral change, the spotlight is turning to the impact of the intra-uterine environment and epigenetics on future risk in adult life. It highlights the urgency for discovering novel approaches to prevention focusing on maternal and child health. Diabetes risk through epigenetic changes can be transmitted inter-generationally thus creating a vicious cycle that will continue to feed the diabetes epidemic. History provides important lessons and there are lessons to learn from major catastrophic events such as the Dutch Winter Hunger and Chinese famines. The Chinese famine may have been the trigger for what may be viewed as a diabetes “avalanche” many decades later. The drivers of the epidemic are indeed genes and environment but they are now joined by deleterious early life events. Looking to the future there is the potential scenario of future new “hot spots” for type 2 diabetes in regions e.g., the Horn of Africa, now experiencing droughts and famine. This is likely to occur should improved economic and living conditions occur over the next few decades. Type 2 diabetes will remain one of the greatest challenges to human health for many years to come.

## Background

In this lecture honoring Professor Stefan S. Fajans of the University of Michigan, I am going to address four major topics related to diabetes:

[a] Statistics about the global diabetes epidemic : facts and fallacies;
[b] Epidemiological data about the diabetes epidemic: secular rises and falls;
[c] The drivers of the type 2 diabetes epidemic; and
[d] Epigenetics and early life exposure of the fetus that may influence the risk of diabetes in adult life.

The Black Death was one of the most devastating pandemics in human history, killing as much as 20% of the world’s population in the 14th century [1]. But that was then. In the 21st century, the question may be asked, is type 2 diabetes the biggest epidemic in history? I believe it is a much bigger epidemic than the Black Death and in this talk I will try to prove it to you.

### Statistics about the global diabetes epidemic: facts and fallacies

The International Diabetes Federation (IDF) has asked a very simple question, if you look at the world’s most populous countries, where would diabetes fit? Numerically diabetes, if it were a nation, would surpass the United States as the third most populated country in the world. While there are approximately 320 million people in the U.S., there are now 415 million people in the world with diabetes according to the IDF [2]. This is clear evidence to suggest that we have a major global problem with type 2 diabetes.

The IDF has attempted to create awareness of the importance of type 2 diabetes. In the year 2000, IDF estimated there were 151 million people with diabetes globally and predicted that by 2030, there would be 324 million people in the world with diabetes [3]. The World Health Organization (WHO) also estimated the global prevalence of diabetes in 2000 and 2030–171 million people with diabetes in 2000 and 366 million by 2030 [4]. They were terribly wrong, because by 2015 there were already 415 million people with diabetes, far above what was predicted in 2000 for 30 years later. And the situation may even be worse than that. To perform its global projections, the IDF estimates how many people have diabetes in each country. If a country does not have data about diabetes prevalence, the IDF extrapolates from another country using regional data [3] or match geography, World Bank income, ethnicity and language [2]. These extrapolations are less reliable. If anything, the current IDF estimates are still a quite serious underestimate.

This issue is of more than academic interest because young researchers in epidemiology may think the IDF and WHO data are gospel. Unfortunately, they may represent a quick grab of data that go out for public relations purposes and not necessarily for public health benefit and planning.

In 1978, a Kroc Foundation International Conference on Epidemiology of Diabetes and Its Macrovascular Complications was held in Santa Ynez Valley, California. Attendees included Kelly West, Peter Bennett, Harry Keen and other legendary figures in diabetes epidemiology. I was also there, though as a “budding” epidemiologist! The Santa Ynez Valley meeting produced a classification of diabetes, diagnostic criteria, and proper protocols for diabetes epidemiology studies [5], so if a study was done in Japan, it would be comparable to one performed in the United States. This heritage of consensus and standardization has been lost of late because of the practice of the IDF and WHO and indeed the Global Burden of Disease Group [6] and others to publish data which underestimate the burden of diabetes and, if used for public health purposes, probably underestimate the resources required to attack the epidemic. So we have issued a word of caution and hopefully the word will get out [6].

The WHO is at least, in part, at fault in this, as they support the STEPS program which diagnoses diabetes based on the fasting glucose level alone [7]. Notably, Stefan Fajans and Jerome Conn did not even include the fasting glucose in their criteria for the oral glucose tolerance test [8]. Whether they were wise or it was an oversight on their part, I cannot say, but we know the measurement of fasting glucose alone underestimates the prevalence of diabetes by 20–25% [9].

If epidemiologists of policy makers use country-specific estimates provided by IDF or WHO for their planning, they should carefully examine the appropriateness of any extrapolation of data from one country to another and evaluate the criteria used to diagnose diabetes. Inappropriate extrapolations and reliance on fasting glucose alone may substantially bias country-specific estimates and adversely impact planning.

Another issue demonstrates the problems researchers have in getting access to primary data sources, especially from the WHO [10]. WHO has historically not released key information related to diabetes and other non-communicable diseases that are vital for public health. We need a lot more transparency from major organizations in helping both researchers and decision makers to understand the true burden of diabetes, and that involves access to the primary data. These are the barriers that we are trying to address now. We should not be using the data put out by WHO or the IDF or even the Global Burden of Disease Research Group [6, 11] uncritically when it comes to public health planning in any country, even the United States.

### Epidemiological data about the diabetes epidemic: secular rises and falls

So what about this epidemic of diabetes? I started my diabetes epidemiology activities in the Pacific [12] and, later on, the Indian Ocean island of Mauritius [13]. The inspiration for me to get into diabetes research came from the early studies of Ian Prior, a famous cardiovascular epidemiologist who, in the mid-1960s, published information about high rates of diabetes in Polynesians living in New Zealand and the Pacific islands [14]. In 1975, I was in London training at Guys Hospital and it was Christmas. It was snowing and cold and no one came to work. I was sitting there by myself and I picked up an old British Medical Journal and read about Ian Prior’s research showing high rates of diabetes in Polynesians. Subsequently Peter Bennett showed that Pima Indians living in Arizona had the highest prevalence of diabetes in any community in the world [15]. There was also a study in Australia showing how indigenous people have high rates of diabetes [16]. So when I returned to Australia from London, I decided to investigate the issue of diabetes in Pacific Island populations.

Our group “swept” through the Pacific and found some of the highest rates of diabetes that had ever been reported [12]. This was a warning to me that we were going to face a diabetes epidemic. Indeed when we did our first Pacific survey in Nauru in 1975, we found a high rate of diabetes – 34.4% in individuals >15 years old [17]. Peter Bennett had reported that >50% of the adult Pima Indian population over the age of 35 years had diabetes [15]. There was clearly a warning there, and indeed, our Melbourne daily newspaper, The Age, published a warning about diabetes as “The Western Killer in Paradise.” Unfortunately, they put my photograph under the headline and it almost killed the point of the story!

After our group showed very high rates of diabetes in the Pacific, the WHO asked me to go to Mauritius to have a look at the diabetes problem there as there were some indications that diabetes was becoming a problem on that beautiful and idyllic Indian Ocean Island. It was important to understand diabetes in Mauritius because although it had a relatively small population of 1.2 million, the population represented three major ethnic groups: Asian Indians from India, Creole- South African Black population, and Chinese people [13]. Together, these three ethnic groups represent approximately two-thirds of the world’s population. Whatever was happening in Mauritius could be extrapolated to other countries where there were Indian, Chinese and Creole or Black populations.

**Fig. 1:**
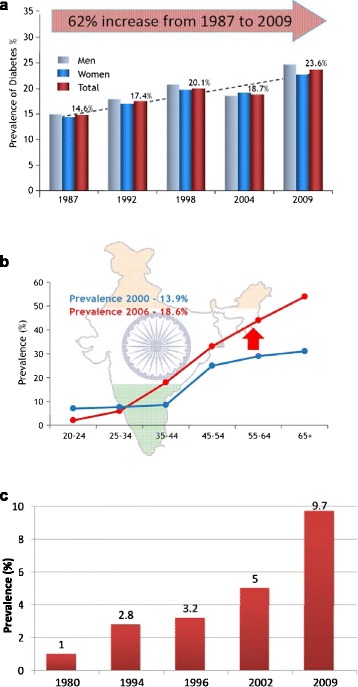
The Increase in Diabetes Prevalence in **a** Mauritius Adapted from [18, 19], **b** India Adapted from [20], and **c** China Adapted from [21]

With a team including Sir George Alberti, a great bio-chemist; Jaakko Tuomilehto, whose name is synonymous with the prevention of type 2 diabetes; my colleagues from Australia, Jonathan Shaw and Dianna Magliano; and Sudhirsen Kowlessur from Mauritius, we have surveyed the population of Mauritius every 5 years or so from 1987 to 2015. Figure 1 shows data from Mauritius from the first study in 1987 to the most recent published study from 2009 [18, 19]. As you can see, the prevalence of diabetes went from 14.6 to 23.6%, a 62% increase over 20 years (Fig. 1a). The pattern was very similar in each ethnic group. Based on these data, we concluded that we were facing a global epidemic of type 2 diabetes, especially in countries such as China and India.

**Fig. 2:**
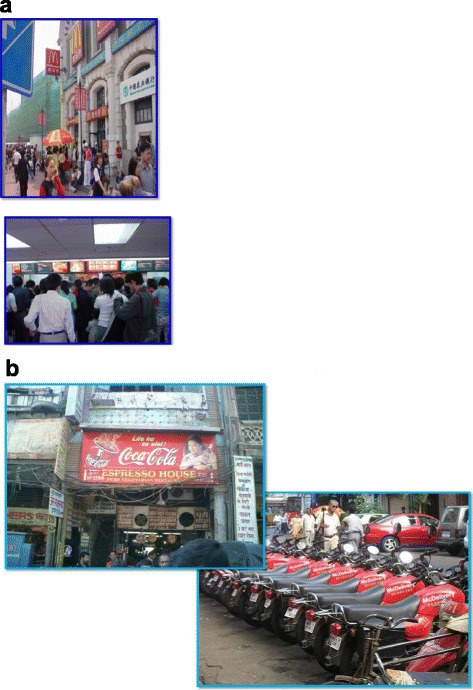
“Coca-colonization” in China and India. **a** McDonald's in China **b** Coca-cola in India

This has now been borne out. In India there has been an increase of nearly 5% (absolute) in diabetes prevalence between the years 2000 and 2006 [20] (Fig. 1b). India now has 80–90 million people with diabetes. In India and in many middle and low-income countries, there are simply not the resources to manage diabetes. In 1980, less than 1% of Chinese population had diabetes. In Beijing, the McDonalds restaurant in Tiananmen Square was one of the busiest McDonald franchise in the world (Fig. 2). Now the estimate of diabetes prevalence in China is 9.7% [21] (Fig. 1c). A study from Turkey has also documented an incredible 90% increase in diabetes prevalence over 11 years from 2002 to 2013 [22].

Indigenous populations are disproportionately affected by diabetes and its complications. In Australia, Indigenous populations residing in the Northern Territory have one of the highest rates of diabetes in the world and certainly among Indigenous communities [23]. Indigenous people in Australia have a 4-fold higher diabetes prevalence compared to the general Australian, mainly European population. They also have the one of the highest rates of end-stage renal disease in the world [24]. Alice Springs, in the center of Australia and the home to a large Indigenous population, has the largest kidney dialysis unit in the southern hemisphere per capita, another reflection of the impact of diabetes [25]. Diabetes also impacts survival. The prevalence of diabetes falls off in the Indigenous population over 64 years of age, not because there is a decrease in the incidence of diabetes, but because of higher mortality in those with diabetes in that age category. [26].

### The drivers of the type 2 diabetes epidemic (Table 1)

**Table 1:** Reported drivers of diabetes

Lifestyle	
Inactivity	
Caloric excess	
Obesity	
Ageing	
Modernization	
Fetal Programming	

In indigenous communities in Australia, as in the US and Canada, it is very important to understand the attitudes of affected people before trying to prevent or manage diabetes. We have a “western” view that diabetes is caused by bad behavior, too much sugary drinks, and eating too much. Obesity and not enough exercise are the culprits. In contrast, the Indigenous people of Australia have lost their lands, are in disharmony with other communities, suffer from poverty and other external pressures (Table 2). Recognizing and addressing these issues is one of the greatest challenges we have in trying to prevent diabetes in the communities of Australia that have the highest rates.

**Table 2:** Indigenous communities: drivers of type 2 diabetes

“Western” view	Indigenous view
Bad behaviors	Dispossession of lands
Bad choices	Disharmony/Imbalance
Lazy	Poverty
Obesity	Socio-cultural change
	“Toxic” external pressures
	Transgenerational trauma

With permission from Professor Alex Brown

**Fig. 3:**
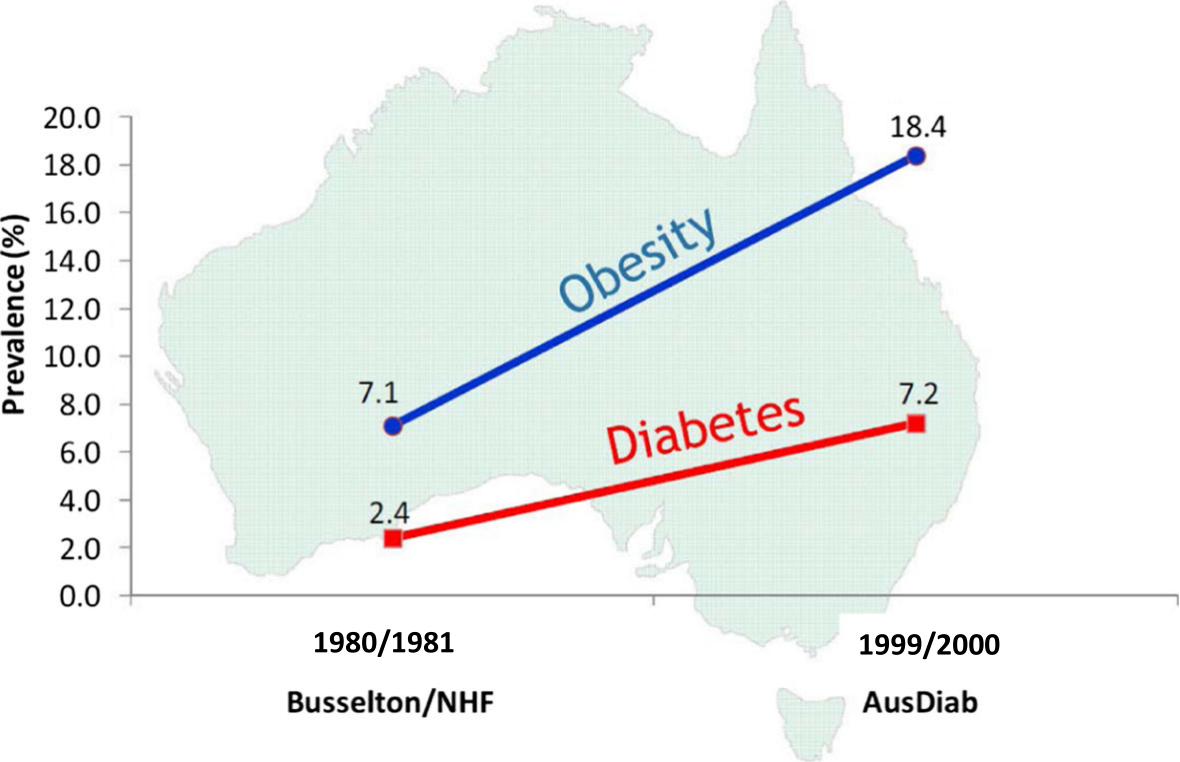
Prevalence of Diabetes and Obesity in Australia 1980/81 and 1999/2000 Adapted from [27, 28]

There are clear links among lifestyle, inactivity, ageing, obesity, and modernization, that contribute to diabetes. Between 1980, when the first diabetes study was done in Australia, and 2000, the prevalence of obesity almost tripled and the prevalence of diabetes increased from 2.4 to 7.2% [27] (Fig. 3). If you look at the incidence of diabetes in the Australian cohort from the year 2000, there was a 4-fold difference in risk of developing diabetes between people who were obese and those who were of normal weight [28]. Almost two-thirds of the Australian population in 2000 was overweight or obese, close to the rates of overweight and obesity in Americans.

So far, I’ve given you all the bad news about the epidemic of diabetes. The question is, are there any studies that show that the prevalence of diabetes is actually falling? Some recent data from the United States now suggest there might be a leveling off of obesity and diabetes [29]. There’s some debate as to why this may be happening; whether it is due to improved public awareness or whether everyone who is going to get diabetes already has it. In Nauru in 1994, the prevalence of diabetes was approximately 50%. In a subsequent study about 10 years later, the rate had fallen [30]. Over the same time period, Nauru went from being the wealthiest country in the world per capita (due to rich phosphate deposits) to one of the poorest countries in the world. So the decline in prevalence could have been an effect of the economic collapse. Another study reported in the British Medical Journal found that during an economic crisis in Cuba, there was a decrease in obesity and a decrease in diabetes incidence and deaths from diabetes [31]. So there’s some evidence that economic hardship may be the best way to prevent diabetes!

The prevention of type 2 diabetes is a major global public health challenge that we now face. Over 20 years ago, a study by the late Hilary King, an adventurous young epidemiologist, found a 4% diabetes prevalence on a small Island off of the coast of Papua New Guinea [32]. This was quite high for Papua New Guinea. We considered doing a prevention study there, but an earthquake occurred and the whole island disappeared. I did not think it was a good way to prevent diabetes, but it solved the problem there!

In 1982, Kerin O’Dea, one of our very well-known Australian diabetes investigators, took a group of Indigenous bush people for 7 weeks to live using traditional foods, such as crocodile, kangaroo and native plants [33]. They lost weight and their glucose tolerance, insulin sensitivity, blood lipids, and blood pressure all improved (Table 3). This was one of the first demonstrations that if you return to a traditional lifestyle, you can reverse not just diabetes but other components of the metabolic syndrome. These results were extended by others including the classic study of Jaakko Tuomilehto, the Finnish Diabetes Prevention Study [34]. He observed a reduction of 58% in the risk of progression to type 2 diabetes; this now seems to be an accepted outcome now among persons at high-risk people of diabetes.

**Table 3:** Impact of 7 weeks of Back to Traditional Hunter Gatherer Lifestyle Change in Australian Aborigines on type 2 diabetes

Weight loss	
Striking improvement in glucose tolerance	
Improved insulin response	
Normalization of blood lipids	
Reduction in blood pressure	

Adapted from [33]

### Epigenetics and risk for diabetes

Although I have always been a strong believer in the “CocaColonization” story, a term suggested by Arthur Koestler [35], that changes of lifestyle in rural and traditional island populations have caused the epidemics of obesity and diabetes, I think there are emerging data that suggest we need to rethink the story and consider the impact of epigenetics. In 1990, David J. P. Barker first proposed that in utero metabolic adaptation defines a trajectory of growth that prepares the fetus for its likely adult environment [36]. What happens in utero to the fetus depends on the mother’s and the father’s behaviors before conception and the mother’s during pregnancy. The story goes back to the Dutch winter famine [37]. At the end of World War 2, there was a famine during the Nazi Germany occupation of Holland. Women who were pregnant were on very poor diets. Some 30 years later, researchers looked to see what happened to the children who were born at that time. They found high rates of diabetes, obesity, hypertension, and indeed some mental disorders like schizophrenia in the offspring of women who were undernourished during early pregnancy. This raised the issue of the famine and what happened many years later when these children became adults. Their risks of chronic diseases were increased.

**Fig. 4:**
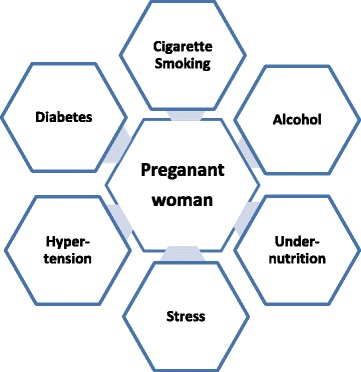
Developmental plasticity, fetal programming and intergenerational risk

Another example occurred during the Chinese famine of 1958–1962 [38]. There was virtually minimal diabetes in China before 1980 [39]. Some 30–40 years after that famine, there are now over 120 million Chinese with diabetes [40]. Again, this raises a question of the role of a famine and the effect of the famine on children exposed to intrauterine undernutrition. In very simple terms, epigenetics reflects not a change in the genes of the fetus, but a change in the DNA around the genes. That DNA influences how the gene reacts with potential environmental risk factors including those noted in the figure (Fig. 4). This can also happen to children born during a famine so that 20–30 years later, when they come into an obesogenic environment, they get diabetes.

**Fig. 5:**
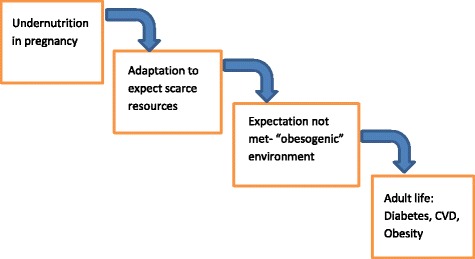
Mismatch: The relevance for prevention of type 2 diabetes

This is just an example of some of the risk factors that can influence *in utero* this process and it’s been well demonstrated in animal studies that this happens. All of the data so far in humans is retrospective, but there is now a prospective study in Singapore led by Professor Sir Peter Gluckman, to assess the impact of epigenetics at a human level [41]. What is also interesting is that diabetes itself is one of the many factors that can cause epigenetic changes. We know that mothers with pre-gestational diabetes and mothers with gestational diabetes are more likely to have offspring who are either obese or have diabetes. And the epigenetic effect appears to be intergenerational. It means you could have a vicious cycle perpetuating the diabetes epidemic. Gluckman and Hansen have authored a book, Mismatch [42], suggesting that a baby born in a famine situation expects to come out into the famine, but may arrive into an obesogenic environment. I have tried to make this phenomenon a little clearer by pointing out that with undernutrition in pregnancy, the adaptation is to expect a scarce resource environment. If the expectation is not met because the baby arrives to an obesogenic environment, we may see both early changes in the child, and an increased risk of obesity, diabetes, and heart disease in adult life (Fig. 5).

I think this is a real story. Higher rates of diabetes also may be linked to famine situations that occurred in the Ukraine (1932–1933) [43] and in Cambodia (1975–79) [44, 45]. We now have a famine in the Horn of Africa. This raises very important issues as to how the United Nations (UN), the WHO, and the UN Development Program and other NGOs handle food relief during and after a famine. These may be very important aspects of preventing diabetes in communities many, many years hence. So finally the message is out to be very wary of national and international predictions of this diabetes epidemic. We need to be looking more closely at maternal and child health, and the whole issue of early development in utero to reduce the risk to future generations. The next epidemic may occur in countries in the Horn of Africa if we do not pay attention to the correct way of handling the nutritional and social issues particularly with aid and food supplies.

## Conclusion

The “Diabesity” epidemic (obesity and type 2 diabetes) is likely to be the biggest epidemic in human history. Diabetes has been seriously underrated as a global public health issue and the world can no longer ignore “the rise and rise” of type 2 diabetes. Currently, most of the national and global diabetes estimates come from the IDF Atlas. These estimates have significant limitations from a public health perspective. It is apparent that the IDF have consistently underestimated the global burden. More reliable estimates of the future burden of diabetes are urgently needed. To prevent type 2 diabetes, a better understanding of the drivers of the epidemic is needed. While for years, there has been comprehensive attention to the “traditional” risk factors for type 2 diabetes i.e., genes, lifestyle and behavioral change, the spotlight is turning to the impact of the intra-uterine environment and epigenetics on future risk in adult life. It highlights the urgency for discovering novel approaches to prevention focusing on maternal and child health. Diabetes risk through epigenetic changes can be transmitted inter-generationally thus creating a vicious cycle that will continue to feed the diabetes epidemic. Yes, diabetes is the greatest epidemic in human history. It has affected the greatest numbers, it has had the greatest cost [46], and it is not over yet.

## Abbreviations

IDF: International Diabetes Federation
STEPS: STEPwise approach to Surveillance
UN: United Nations
US: United States
WHO: World Health Organization
